# The genome of *Haberlea rhodopensis* provides insights into the mechanisms for tolerance to multiple extreme environments

**DOI:** 10.1007/s00018-024-05140-3

**Published:** 2024-03-05

**Authors:** Saurabh Gupta, Veselin Petrov, Vanika Garg, Bernd Mueller-Roeber, Alisdair R. Fernie, Zoran Nikoloski, Tsanko Gechev

**Affiliations:** 1 Intercellular Macromolecular Transport, Max Planck Institute of Molecular Plant Physiology, Am Mühlenberg 1, 14476 Potsdam-Golm, Germany; 2 Curtin Medical School, Curtin Health Innovation Research Institute (CHIRI), Curtin University, Perth, WA 6102 Australia; 3 Center of Plant Systems Biology and Biotechnology, 14 Knyaz Boris I Pokrastitel Str., 4023 Plovdiv, Bulgaria; 4 Department of Plant Physiology, Biochemistry and Genetics, Agricultural University Plovdiv, 12 Mendeleev Str., 4000 Plovdiv, Bulgaria; 5 Molecular Biology, Institute of Biochemistry and Biology, University of Potsdam, Karl-Liebknecht-Str. 24-25, 14476 Potsdam-Golm, Germany; 6 State Agricultural Biotechnology Centre, Centre for Crop and Food Innovation, Food Futures Institute, Murdoch University, Murdoch, WA 6150 Australia; 7 Plant Signalling, Max Planck Institute of Molecular Plant Physiology, Am Mühlenberg 1, 14476 Potsdam-Golm, Germany; 8 Central Metabolism, Max Planck Institute of Molecular Plant Physiology, Am Mühlenberg 1, 14476 Potsdam-Golm, Germany; 9 Bioinformatics, Institute of Biochemistry and Biology, University of Potsdam, Karl-Liebknecht-Str. 24-25, 14476 Potsdam-Golm, Germany; 10 Systems Biology and Mathematical Modelling, Max Planck Institute of Molecular Plant Physiology, Am Mühlenberg 1, 14476 Potsdam-Golm, Germany; 11 Department of Plant Physiology and Molecular Biology, Plovdiv University, 24 Tsar Assen Str., 4000 Plovdiv, Bulgaria

**Keywords:** Resurrection species, Combined abiotic stresses, Stress adaptation, Genome assembly, Transcriptomic profiling, Extremophile

## Abstract

*Haberlea rhodopensis*, a resurrection species, is the only plant known to be able to survive multiple extreme environments, including desiccation, freezing temperatures, and long-term darkness. However, the molecular mechanisms underlying tolerance to these stresses are poorly studied. Here, we present a high-quality genome of Haberlea and found that ~ 23.55% of the 44,306 genes are orphan. Comparative genomics analysis identified 89 significantly expanded gene families, of which 25 were specific to Haberlea. Moreover, we demonstrated that Haberlea preserves its resurrection potential even in prolonged complete darkness. Transcriptome profiling of plants subjected to desiccation, darkness, and low temperatures revealed both common and specific footprints of these stresses, and their combinations. For example, *PROTEIN PHOSPHATASE 2C* (*PP2C*) genes were substantially induced in all stress combinations, while *PHYTOCHROME INTERACTING FACTOR 1* (*PIF1*) and *GROWTH RESPONSE FACTOR 4* (*GRF4*) were induced only in darkness. Additionally, 733 genes with unknown functions and three genes encoding transcription factors specific to Haberlea were specifically induced/repressed upon combination of stresses, rendering them attractive targets for future functional studies. The study provides a comprehensive understanding of the genomic architecture and reports details of the mechanisms of multi-stress tolerance of this resurrection species that will aid in developing strategies that allow crops to survive extreme and multiple abiotic stresses.

**Supplementary Information:**

The online version contains supplementary material available at 10.1007/s00018-024-05140-3.

## Introduction

*Haberlea rhodopensis* Friv. from the Gesneriaceae family belongs to the resurrection species, a small group of plants that can tolerate water loss to air-dried state (i.e., complete desiccation) and regain normal turgor and physiological activities upon rehydration [1, 2]. There are even fewer plant species, confined to a few Gymnosperms, that can tolerate long-term darkness, and no studied land plant except *H. rhodopensis* can tolerate both stresses simultaneously [3, 4]. This resurrection plant can also tolerate freezing temperatures during the winters in its natural habitat, and high levels of oxidative stress [1, 5, 6]. Yet, despite these unique features, the molecular mechanisms that enable *H. rhodopensis* to cope with combined stresses remain poorly explored, although they may provide new strategies to improve climate resilience of agriculturally relevant crops.

The genetic repertoire of *H. rhodopensis*, a diploid species, is unknown due to the unavailability of its genome sequence. However, advances in genome sequencing and assembly techniques have opened the doors for high-quality assemblies. Recently, complementary methodological approaches offered by Pacific Biosciences (PacBio), Oxford Nanopore, BioNano Genomics, and others, as well as chromosome conformation capture sequencing (Hi-C), have gained popularity and have been used to develop high-quality genome assemblies of different plant species [7–11].

To unravel the genetic basis of the unique multi-stress tolerance of *H. rhodopensis*, we report a high-quality de novo sequencing, assembly, and analysis of its genome. In addition, we performed a comparison with the genomes of 20 other land plant species, including various model and resurrection species, to identify gene families expanded in and specific to *H. rhodopensis*. To understand the molecular mechanisms of multi-stress tolerance, we performed transcriptional profiling during several extreme stresses and their combinations. Altogether, the comprehensive analysis presented in the study delineated the unique genome and transcriptome features of *H. rhodopensis* that allow it to withstand individual and combined extreme environmental conditions.

## Materials and methods

### Plant material, growth, stress treatments, and RNA extraction

The initial *H. rhodopensis* Friv. plants were collected from the Rhodopi mountain near Assenovgrad, Bulgaria (location: 24° 52′ E, 41° 55′ N; elevation 690 m), and their in vitro culture was established. *H. rhodopensis* plants were grown and propagated under optimal conditions (16/8 h light/dark photoperiod, 35 µE m^−2^ s^−1^ light intensity, 20 °C), as previously described [3]. For darkness experiments, in vitro cultivated plants at the rosette leaf stage were divided into groups and subjected to three stress conditions: desiccation (until the relative water content (RWC) reached 5%), complete darkness for one month, and a combination of the two stress factors (desiccation in darkness). Well-hydrated plants had an RWC of about 85%. Desiccation to an air-dry state was conducted by removing the growth media for seven days at room temperature and 70% relative humidity until plants reached an air-dried state at RWC of 5%. Plants were rehydrated for five days by returning them to well-hydrated media until reaching the original RWC. For dark treatments, plants were subjected to darkness for one month, followed by a recovery period of seven days under normal light conditions. For recovery period, the plants desiccated in darkness were rehydrated in the dark, while those that were desiccated in the light were rehydrated in the light. Rosette leaves were collected in a dark room illuminated with non-actinic green light and immediately frozen in liquid nitrogen. In parallel, groups of control plants were maintained at normal conditions and were taken along the experiment with the respective time points to provide proper developmental controls.

Control plants, as well as plants subjected to desiccation (5% RWC), one-month darkness, the combination of the two stress factors (desiccation in darkness), and plants that have recovered from the stress conditions, were used to isolate and purify RNA for RNA-seq as described previously [1]. Samples were obtained in three biological replicates. The details of samples used for transcriptome sequencing are provided in Table S1. The isolated RNA was subjected to RNA-seq using an Illumina HiSeq X Ten by BGI Tech Solutions, Tai Po, Hong Kong.

For the low temperatures experiments, in vitro cultivated plants at the rosette leaf stage were transferred to Petri dishes with the following soil: Rėkyva Remix Fine peat substrate and agro perlite in 2.5:1 ratio, supplemented with a fertilizer mix containing 1.2 g Ca(H_2_PO_4_)_2_, 0.5 g K_2_SO_4_, 0.5 g NH_4_NO_3_ and 0.2 g MgSO_4_ per 1 L of potting mix. Thereafter, plants were cultivated in the same conditions as described above for at least three additional months to allow them to adapt to the new environment. At the onset of the experiment, 120 plants were divided into two populations: one group was left without water for 18 days (until plants reached ~ 10% RWC), while irrigation was retained for the other one. Then, each set was divided further into four subpopulations based on the temperature treatment: standard temperature, 24 h chilling (4 °C), 24 h freezing (− 4 °C), and chilling followed by recovery (also for 24 h) and subsequent freezing. Thus, eight experimental groups were obtained, each consisting of 15 plants used to generate three independent pools (5 plants per pool). Leaves were flash-frozen in liquid nitrogen and used for further physiological and transcriptomic analyses. RNA was extracted by Zymo Research Quick-RNA Miniprep kit, following the manufacturer’s instructions, with a few modifications as follows: inclusion of an incubation step with the lysis buffer at room temperature for 30 min; doubling the number of washing steps with RNA wash buffer (to remove impurities from *H. rhodopensis* leaves); reduction of the utilized ground leaf tissue to ~ 10–15 mg; and a slight prolongation of the centrifugation times. The isolated RNA was subjected to RNA-seq using the DNBseq™ sequencing technology by BGI Tech Solutions.

### Measurements of electrolyte leakage and relative water content

Electrolyte leakage was assessed by measuring the increase in conductivity with an HI 873 conductivity meter (Hanna Instruments, Woonsocket, RI, USA). Haberlea leaves were briefly washed with ultrapure water (conductivity of 1 µS). The leaves were then incubated in ultrapure water (1 µS) for 10 min. The conductivity of the resultant solution was measured and compared with the total conductivity obtained after boiling the leaves. RWC was determined using the formula RWC (%) = [(FM − DM)/(TM − DM)], where FM, DM, and TM are the fresh, dry, and turgid masses of the leaves, respectively. TM was measured after immersing the leaves in H_2_O for 24 h, and DM was determined after drying the leaves at 80 °C for 48 h. RWC was determined in three biological replicates.

### Library preparation and genome sequencing

High-molecular-weight genomic DNA was prepared from leaves using a custom-designed protocol established at the Massey NGS facility, Massey University, New Zealand [12]. Genomic DNA was used to generate a 10–20 kb sequencing library according to the instructions of Pacific Biosciences (PacBio). The library was sequenced with a PacBio Sequel II instrument using 24 SMRT cells to generate ultra-long reads by DNA Link Inc., Seodaemun-gu, Seoul, Korea. Additionally, the genomic DNA was used to generate a Dovetail HiC library as per the manufacturer’s protocol, which was further sequenced on an Illumina platform using a paired-end strategy (Dovetail Genomics, CA, USA) (Data S1).

### Genome assembly and assessment

The long reads from PacBio sequencing were assembled using FALCON-Unzip [13] with default parameters and Canu v1.7 [14] with parameters tuned for heterozygous genomes. Due to the high heterozygosity of the *H. rhodopensis* genome (1.48%; estimated using GenomeScope available at http://qb.cshl.edu/genomescope/; [15]), the following Canu parameters were used “corMhapSensitivity = normal corOutCoverage = 200 correctedErrorRate = 0.105 "batOptions = -dg 3 -db 3 -dr 1 -ca 500 -cp 50"”. The assemblies obtained were polished using one round of Arrow (SMRT Link v5.1, https://www.pacb.com/support/software-downloads/) with default parameters. The Arrow-polished Canu assembly was processed to obtain primary contigs using Purge Haplotigs v1.1.1 [16] with default parameters. The Canu-de-duplicated and FALCON-Unzip assemblies were scaffolded using the HiC reads utilizing the SALSA pipeline v2.2-14-g974589f [17]. The assembly statistics were generated using QUAST v4.1 [18]. Additionally, the completeness of assemblies was evaluated using BUSCO v5.4.4 [19],"viridiplantae_odb10" dataset). Based on the assembly and completeness statistics, Canu + SALSA assembly was deemed the best assembly and was used for downstream analysis (Fig. S1).

### Repeat identification

Both de novo and homology-based repeat identification approaches were used to identify and annotate repeats in the *H. rhodopensis* genome. First, a de novo repeat library was constructed using RepeatModeler version open-1.0.10 with default parameters [20]. The de novo repeat library obtained was combined with the known Viridiplantae-based repeats from RepBase version 20170127 [21] to generate a custom repeat library. This library was then used to screen the genome for repeats using RepeatMasker version open-4.0.7 (“-u -gff -e ncbi -xsmall” [22]).

### Gene prediction and annotation

The raw reads from RNA-seq datasets were trimmed to remove low-quality bases and sequencing adaptors using Trimmomatic v0.39 [23]. The trimmed reads were filtered for ribosomal RNA using SortMeRNA v2.1 [24]. The filtered RNA-seq reads were aligned against the genome assembly using HISAT2 v2.1.0 [25] and assembled into individual transcripts using StringTie2 v2.0 [26], which were merged to obtain a consensus assembly. Further, the ab initio gene model predictions were obtained by the BRAKER2 pipeline v2.1.5 [27] using evidence from RNA-seq, de novo transcriptome assembly [3], and homology-based alignments with known plant proteins from SwissProt. Finally, EVidenceModeler v1.1.1 [28] was employed to combine RNA seq-based predictions with ab initio gene predictions to obtain a final set of gene models. Functional annotation to the predicted genes was performed using similarity searches against various publicly available databases. Briefly, BLASTP (E-value 1E-05) was used to search against the NCBI non-redundant (NCBI-nr), SwissProt, and TrEMBL databases with taxonomy filter for "Viridiplantae" (taxonomy id: 33090). The BLAST annotations obtained from these databases were merged to assign functional annotation to protein sequences (Swissprot preferred over TrEMBL and then NCBI nr). Mercator4 V2.0 [29] was used to obtain MapMan4 annotations for the predicted proteins. InterProScan v5.39–77.0 [30] was used to identify conserved domains and motifs in the proteins encoded by gene models. Gene Ontology IDs for each gene were obtained from the corresponding InterPro entry. Further, rRNAs, miRNAs, and snRNAs, were predicted by homology searches against the Rfam database (release 14.2) using Infernal v1.1.3 [31] with default parameters. The tRNA genes in the genome were identified by tRNAscan-SE v2.0 [32] with default parameters. The non-coding RNA encoding genes for *Craterostigma plantagineum* and *Lindernia brevidens* were also predicted using Infernal and tRNAscan.

### Gene family and synteny analysis

The predicted protein sequences from *H. rhodopensis* were compared against protein sequences of 20 other plant species using OrthoFinder v2.4.0 [33] to identify sets of orthologous genes, referred to as gene families. Single-copy orthologs obtained were used to construct the phylogenetic tree. The expansion/contraction analysis was performed by CAFE v4.2.1 (parameters: “-p 0.01 -t 4 -r 10,000 -filter” [34]) based on the species tree and gene family statistics. Based on this ortholog analysis, the genes that are specifically present in *H. rhodopensis* are classified as “orphan genes”. The significantly expanded gene families (FDR < 0.05) in *H. rhodopensis* were manually annotated based on the annotation of homologs from Arabidopsis or InterProScan annotations. The *ELIP* gene family members were manually curated to check for presence of PF00504 domain by searching against Pfam using InterProScan. Divergence times between *H. rhodopensis* and other plant species were estimated using MegaX software (https://www.megasoftware.net/) with default parameters using the known calibration times from the TimeTree database (http://www.timetree.org, [35]). The Synteny analysis was performed using GENESPACE R package (v1.2.3 [36]).

### Transcription factors and resistance gene analogs

Transcription factors (TFs) in the *H. rhodopensis* genome were identified using a similarity search against plant transcription factors from the plant transcription factor database version 5.0 [37]. The RGAugury pipeline v2017-10-21 with default parameters was used to identify resistance gene analogs (RGAs) from the predicted gene set of *H. rhodopensis* [38]. The identified RGAs were then classified into different sub-classes based on the presence or absence of specific domains.

### Transcriptome analysis

The raw reads from RNA-seq datasets were trimmed to remove low-quality bases and sequencing adaptors using Trimmomatic. The trimmed reads were filtered for ribosomal RNA using SortMeRNA. The filtered RNA seq reads were aligned against the genome assembly using HISAT2. The transcript- and gene-level quantifications were obtained using StringTie2. For gene expression analysis, the EdgeR package [39] in R/Bioconductor was used for multiple pairwise comparisons. A false discovery rate (FDR) cutoff (Benjamini–Hochberg correction) of less than 0.05 and a log2 fold change ≥ 1 was used to identify significantly differentially expressed genes. Heat maps and clustering for selected groups of genes were made using the ComplexHeatmap R package [40]. The optimal number of k-means clusters was estimated using XMeans algorithm in the RWeka package [41]. TopGO was used to perform GO enrichment analysis with FDR cutoff of 0.05. The enrichment analysis for MapMan4 annotations was performed using hypergeometric test [42] with a significance threshold of FDR 0.05. The local FDR correction method was used to adjust the *P*-values obtained from GO and MapMan bin enrichment analysis. For gene set enrichment analysis, the genes that had a GO and MapMan annotation were considered as background. For enrichment analysis of the ortho groups, the ortho groups were assigned a GO based on the longest member of each ortho group and the ortho groups that had a GO annotation were used as background.

## Results

### Sequencing and assembly of the *Haberlea rhodopensis* genome

**Fig. 1:**
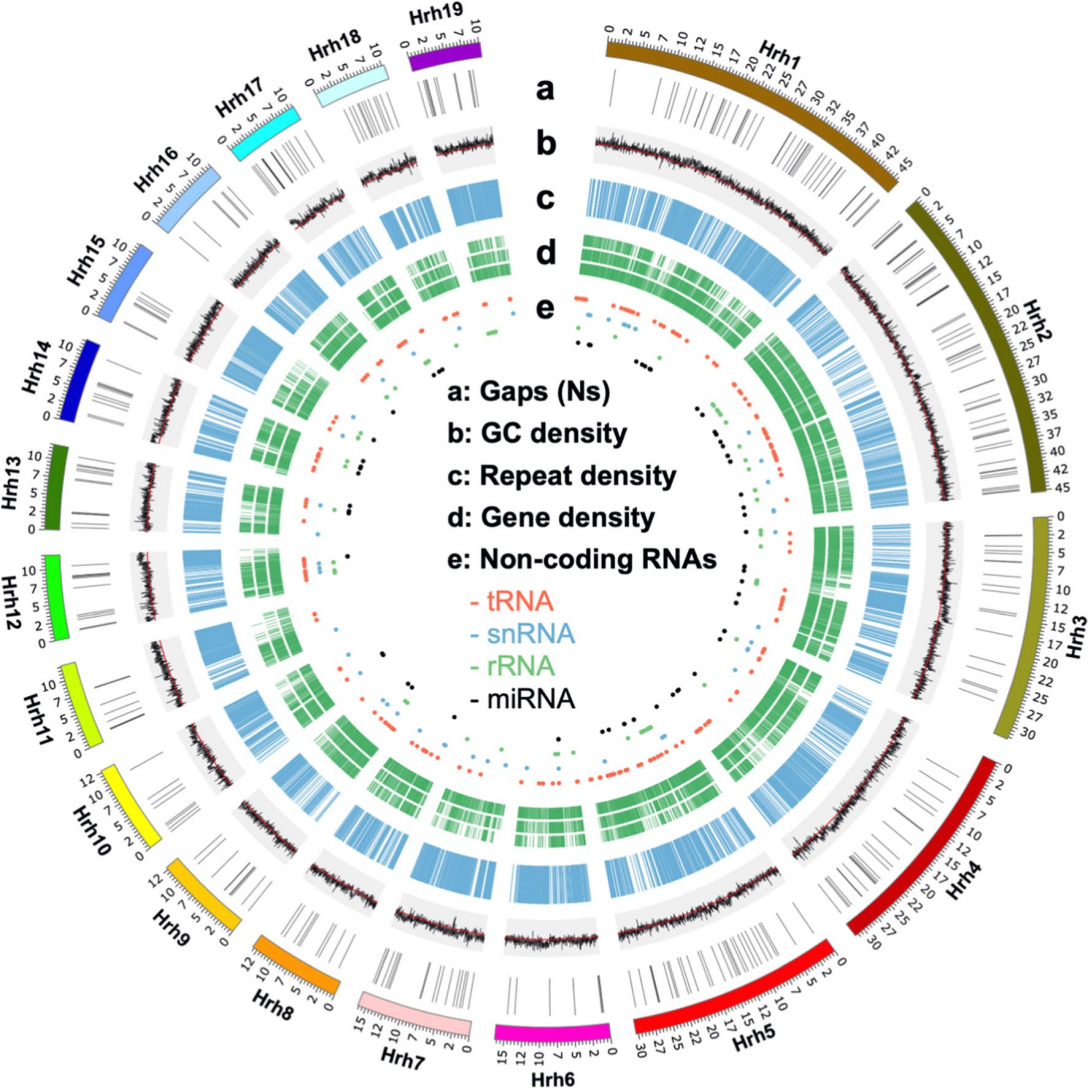
Genomic features of the longest scaffolds in the *H. rhodopensis* genome. Each circular track depicts the distribution of different features of the *H. rhodopensis* genome. **a** Gaps. **b** GC density (bin size: 20 kb; red line indicates average GC content). **c** Repeat density (bin size: 10 kb). **d** Gene density. **e** Distribution of non-coding RNAs including tRNAs (red), snRNAs (blue), rRNAs (green) and miRNAs (black)

Using a combination of PacBio (~ 91X) and HiC sequencing (~ 176 million read pairs), we developed a high-quality ~ 1.27 Gb (N50 = 2.92 Mb) genome assembly of *H. rhodopensis* consisting of 3499 pseudo-scaffolds (Fig. 1; Table 1; Table S2; Data S1; Fig. S1). The assembly accounts for 92.7% of the estimated genome size of ~ 1.37 Gb [43]. Several scaffolds did not attain chromosome lengths possibly due to chimeric read mapping. The GC content of the *H. rhodopensis’* genome (38.08%) is similar to that of *Arabidopsis thaliana* (36%), *Streptocarpus rexii* (38.89%; member of Gesneriaceae family; [44]) and *Xerophyta viscosa* (36.51%), a resurrection species from the Velloziaceae family [45], but lower than that of other resurrection species including *Boea hygrometrica* (42.30%; Gesneriaceae family; [46]), and two from the Linderniaceae family (*C. plantagineum* (40.26%; [47]) and *L. brevidens* (39.26%; [48])) (Fig. S2A).

**Table 1:** Assembly and annotation statistics for the *H. rhodopensis* genome

*Assembly features*	
Total assembly size	1.27 Gb
Total number of scaffolds	3499
Scaffold N50	2.92 Mb
Longest scaffold	45.91 Mb
Number of scaffolds > 1 Mb	216
Number of scaffolds > 10 Mb	19
GC content	38.08%
BUSCO completeness (genome)	99.1%
*Protein-coding genes*	
Number of protein-coding genes	44,306
Mean gene length	4697 bp
Number of transcripts	93,489
Average transcript length	1589.5 bp
Average number of exons per transcript	5.2
Mean exon length	336 bp
Number of annotated genes	39,538
Number of unannotated genes	4768
BUSCO completeness (proteins)	95.8%
*Non-coding genes*	
Number of rRNA fragments	666
rRNA fragments share in genome	413.8 kb
Number of tRNA genes	626
tRNA genes share in genome	47.5 kb
Number of miRNA genes	205
miRNA genes share in genome	27.7 kb
Number of snRNA genes	160
snRNA genes share in genome	21.8 kb
*Transposable elements*	
Total size of transposable elements (TEs)	872.08 Mb
TEs shared in genome	68.68%

### Repeat content and gene annotation

Applying de novo repeat identification, we found that 872.08 Mb (68.68%) of the *H. rhodopensis* genome was repetitive, similar to the repeat content estimated for *B. hygrometrica* [46] and *Solanum lycopersicum* [49]. In accordance with the pattern observed in other plant genomes, long-terminal repeat (LTR) retrotransposons were the most abundant class of repetitive DNA and comprised nearly 41% of the *H. rhodopensis* genome, which was slightly higher than the LTR composition in other resurrection species including *C. plantagineum* (35%) and *L. brevidens* (34%). Among LTR transposons, Gypsy and Copia elements represented 22.88% and 17.09% of the genome, respectively (Table S3). Furthermore, the synonymous mutation rate (Ks) distribution of the paralogs indicated three distinct major duplication events in *H. rhodopensis* that could explain its large genome size and high repeat content (Fig. S2B).

By integrating homology searches, ab initio prediction, and mRNA expression evidence, we predicted a total of 44,306 protein-coding genes in the *H. rhodopensis* genome (Fig. 1). On average, the predicted genes encode transcripts of 1589.5 bp length with 5.2 exons, similar to reports for other plant species (Table 1; [50, 51]). Based on a similarity search against different databases, we annotated 39,538 genes (89.24%, Table S4). We identified 666 genes for ribosomal RNAs (rRNAs), 626 transfer-RNAs (tRNAs), 205 microRNAs (miRNAs), and 160 small nuclear RNAs (snRNAs; Table 1). These numbers were similar to those for *B. hygrometrica* and *L. brevidens*. However, *C. plantagineum* contained significantly higher number of all non-coding genes. Among the rRNA genes, we found 273 5S rRNA genes which is significantly higher than the 5S rRNA genes present in *S. lycopersicum*. Interestingly, a similar observation was reported in *B. hygrometrica*; however, in the case of *H. rhodopensis* 88.3% (241) of these 5S rRNA genes were found in clusters of tandem repeats on three scaffolds which contrasts with *B. hygrometrica* where the majority of them were interspersed throughout the genome. Furthermore, more than 99% of the 425 core Viridiplantae genes are conserved in the *H. rhodopensis* genome assembly, indicating a high-quality assembly. Synteny analysis with the closely related species (*A. thaliana* and *S. lycopersicum*) further suggested the high quality of the Haberlea genome assembly (Fig. S3).

### Comparative analysis of the *H. rhodopensis* genome with those of other plants

**Fig. 2:**
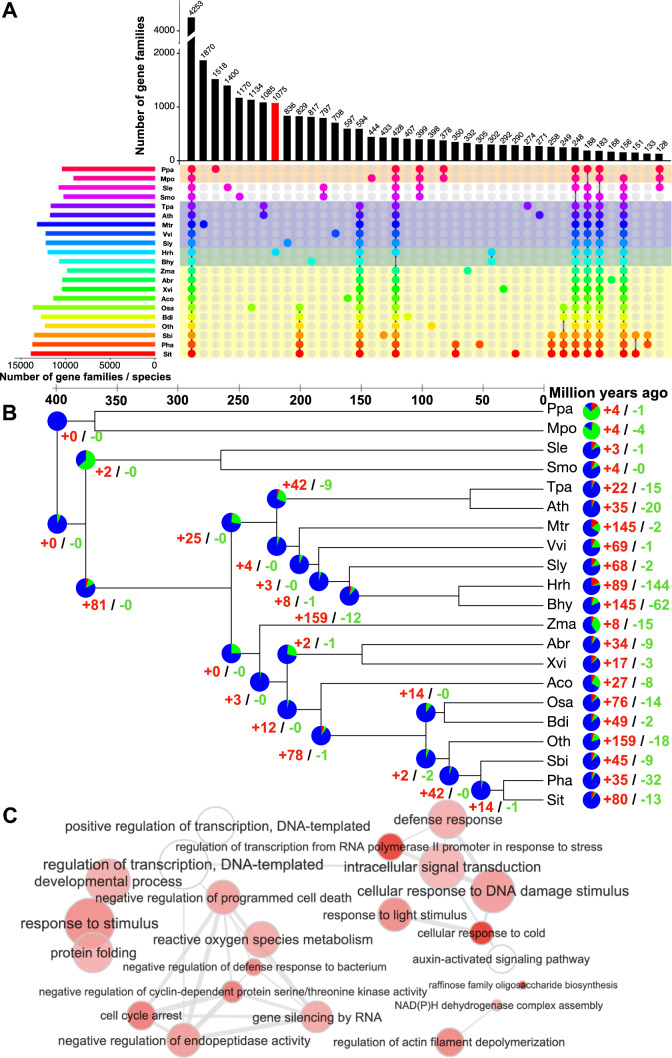
Gene family analysis in comparison to other land plant species. **A** An UpSet plot representation of the shared and unique gene families between 21 plant species. The horizontal bars (left) show the total number of gene families in each species; vertical bars represent the frequency for each intersection (shared or unique gene families), and colored circles highlight the species that are part of the intersection. Altogether 4253 gene families are shared between all 21 species, whereas 1075 are specific to *H. rhodopensis* (red bar). **B** Phylogenetic tree constructed using 82 single-copy orthologs from 21 different plant species (Bootstraps: 1000). The pie charts at the nodes depict the number of gene families expanded (red), contracted (green), and unchanged (blue). The numbers next to the pie charts in red and green represent the number of significantly expanded and contracted gene families, respectively (FDR < 0.01). **C** Significantly enriched biological process gene ontology (GO) terms (FDR < 0.05) for gene families expanded in *H. rhodopensis*. Color intensity reflects the significance of enrichment, with darker colors corresponding to lower FDR. Circle radii depict the size of aggregated GO terms. Data for panel (**C**) are provided in Data S2. Abbreviations; Ppa, *Physcomitrella patens*; Mpo, *Marchantia polymorpha*; Smo, *Selaginella moellendorffii*; Sle, *Selaginella lepidophylla*; Ath, *Arabidopsis thaliana*; Tpa, *Thellungiella parvula*; Mtr, *Medicago truncatula*; Vvi, *Vitis vinifera*; Sly, *Solanum lycopersicum*; Bhy, *Boea hygrometrica*; Hrh, *Haberlea rhodopensis*; Zma, *Zostera marina*; Xvi, *Xerophyta viscosa*; Aco, *Ananas comosus*; Abr, *Acanthochlamys bracteate*; Osa, *Oryza sativa*; Bdi, *Brachypodium distachyon*; Oth, *Oropetium thomaeum*; Sbi, *Sorghum bicolor*; Pha, *Panicum hallii*; Sit, *Setaria italica*

Next, we compared the genes predicted in *H. rhodopensis* with those of 20 other land plant species from evolutionarily divergent groups, including several other resurrection plants, to identify unique and shared gene families. Reciprocal pairwise BLAST comparisons using OrthoFinder grouped 663,415 proteins into 36,943 ortholog clusters (henceforth referred to as gene families) (Table S5). A total of 4253 gene families were present across all 21 species (Fig. 2A). The phylogenetic analysis suggested that Gesneriaceae species diverged from *S. lycopersicum* (Solanaceae) around 100–130 million years (myr) ago, in accordance with previous reports [52], and members of Gesneriaceae, *H. rhodopensis* and *B. hygrometrica* diverged from each other approximately 56 myr ago (Fig. 2B).

Orphan genes are important for taxonomy-specific developmental adaptations [53]. In *H. rhodopensis,* based on ortholog analysis, we found 10,435 orphan genes (23.55% of total genes) which is within the expected range observed in eukaryotes [54]. A total of 1075 gene families were specific to *H. rhodopensis* (Fig. 2A). The highest number of gene families coded for proteins with unknown functions and without any known domains that can suggest biological function. Four of the other families encoded zinc finger proteins (CCHC-type superfamily and SWIM-type, as well as with an integrase zinc-binding domain), suggesting a role in transcription/DNA binding. The other Haberlea-specific gene families include *ULP_PROTEASE DOMAIN-CONTAINING PROTEINS*, an *ASPARTIC PEPTIDASE DOMAIN FAMILY*, and *GUANYLATE-BINDING PROTEIN 4-LIKE FAMILY*.

A total of 89 and 144 gene families in *H. rhodopensis* were significantly expanded and contracted, respectively (Table S6). Of the 89 expanded gene families, 25 were specifically found in *H. rhodopensis*. Some of the more notable expanded gene families include: *ZINC FINGER CCHC DOMAIN FAMILY*, *RAC-LIKE*, *SQUAMOSA PROMOTER BINDING PROTEIN-LIKE*, *SERINE/THREONINE-PROTEIN PHOSPHATASE 7 (PP7) LONG FORM HOMOLOG (PP7L)*, *TCP FAMILY TRANSCRIPTION FACTOR*, *WRKY DNA-BINDING PROTEIN*, *FRS (FAR1-RELATED SEQUENCES) TRANSCRIPTION FACTOR*, *AT-HOOK MOTIF NUCLEAR-LOCALIZED PROTEIN*, and *SWIM-TYPE DOMAIN-CONTAINING PROTEIN.* Furthermore, 159 gene families were expanded in the Gesneriaceae species that included *EARLY LIGHT-INDUCED PROTEINS* (*ELIPs*). ELIPs are known to play a crucial role in desiccation tolerance and are reported to be expanded in other resurrection species as well [55, 56]. Three other gene families that significantly expanded in *H. rhodopensis* contain homologs of the bHLH, WRKY and FRS transcription factor (TF) families. The FRS TFs have been implicated in the regulation of genes associated with drought, salinity, and temperature fluctuations [57, 58]. The expanded gene families were enriched for genes related to ‘auxin activated signaling pathway’, ‘reactive oxygen species metabolic process’, ‘defense response’, ‘cellular response to cold’, ‘flower development’, ‘cellular response to heat’, and ‘protein folding’ (Fig. 2C; Data S2). In terms of MapMan categories, these gene families are involved in diverse functional categories, such as cell wall organization, SnRK1 kinase regulatory system, RNA processing, carbohydrate metabolism, and chromatin organization (Fig. S4). Similarly, the contracted gene families were enriched for genes involved in “cell recognition”, “recognition of pollen” and “proteolysis” (Data S2).

### *H. rhodopensis* can withstand multiple and combined stresses

**Fig. 3:**
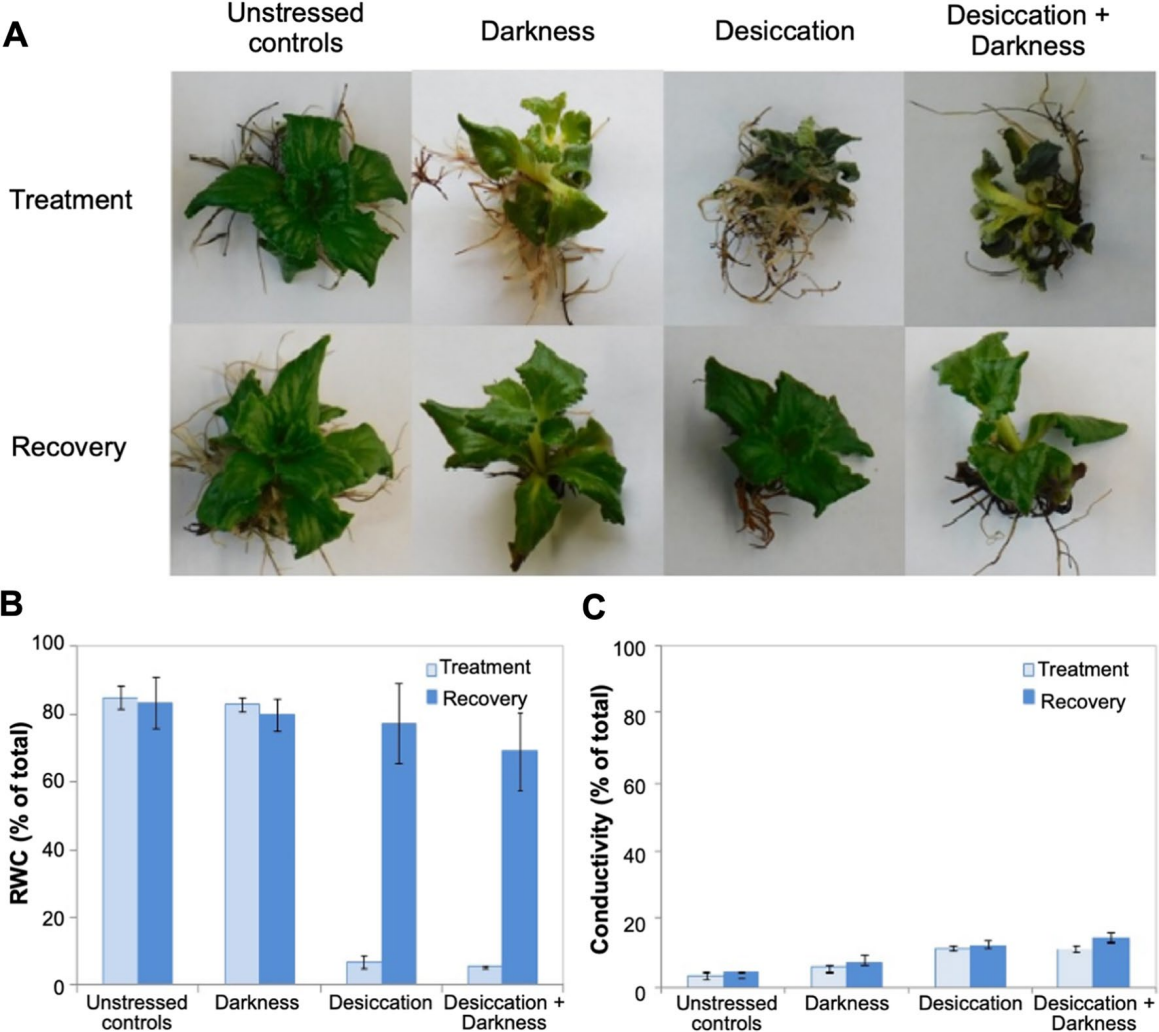
*H. rhodopensis* can tolerate desiccation, darkness, or the combination of the two stresses. **A** Plants were treated with one month of darkness, desiccation until air-dried state (5% relative water content (RWC)), and desiccation in darkness and subsequently returned to optimal growth conditions for full recovery. **B** Relative water content and **C** Electrolyte leakage measured as conductivity for the respective plants. Data are means ± SD of three biological replicates

We previously observed that *H. rhodopensis* withstands desiccation and darkness as individual stresses [3]. To study the combined effect of both stresses, we subjected *H. rhodopensis* plants to desiccation, complete darkness, or the combination of the two, for one month and then returned them for five days to optimal conditions for recovery (Fig. 3A; see Materials and Methods). Plants in darkness were slightly etiolated but preserved most of their chlorophyll, and eventually completely recovered (Fig. 3A). Plants subjected to desiccation in the light became air-dried after seven days and then regained their turgor and relative water content upon rehydration (Fig. 3B). Plants subjected to desiccation in darkness lost their turgor and part of their chlorophyll; however, they fully recovered when returned to optimal growth conditions (Fig. 3A). The desiccated plants (both under normal photoperiod and in darkness) had very low (< 5%) relative water content (RWC), indicating almost complete dehydration. However, cell damage was limited, as revealed by a very low electrolyte leakage (Fig. 3C). Overall, the two stresses given separately, or in combination, led to clear and predictable phenotypic changes; however, all plants fully regained their normal appearance after one week of recovery from stress.

**Fig. 4:**
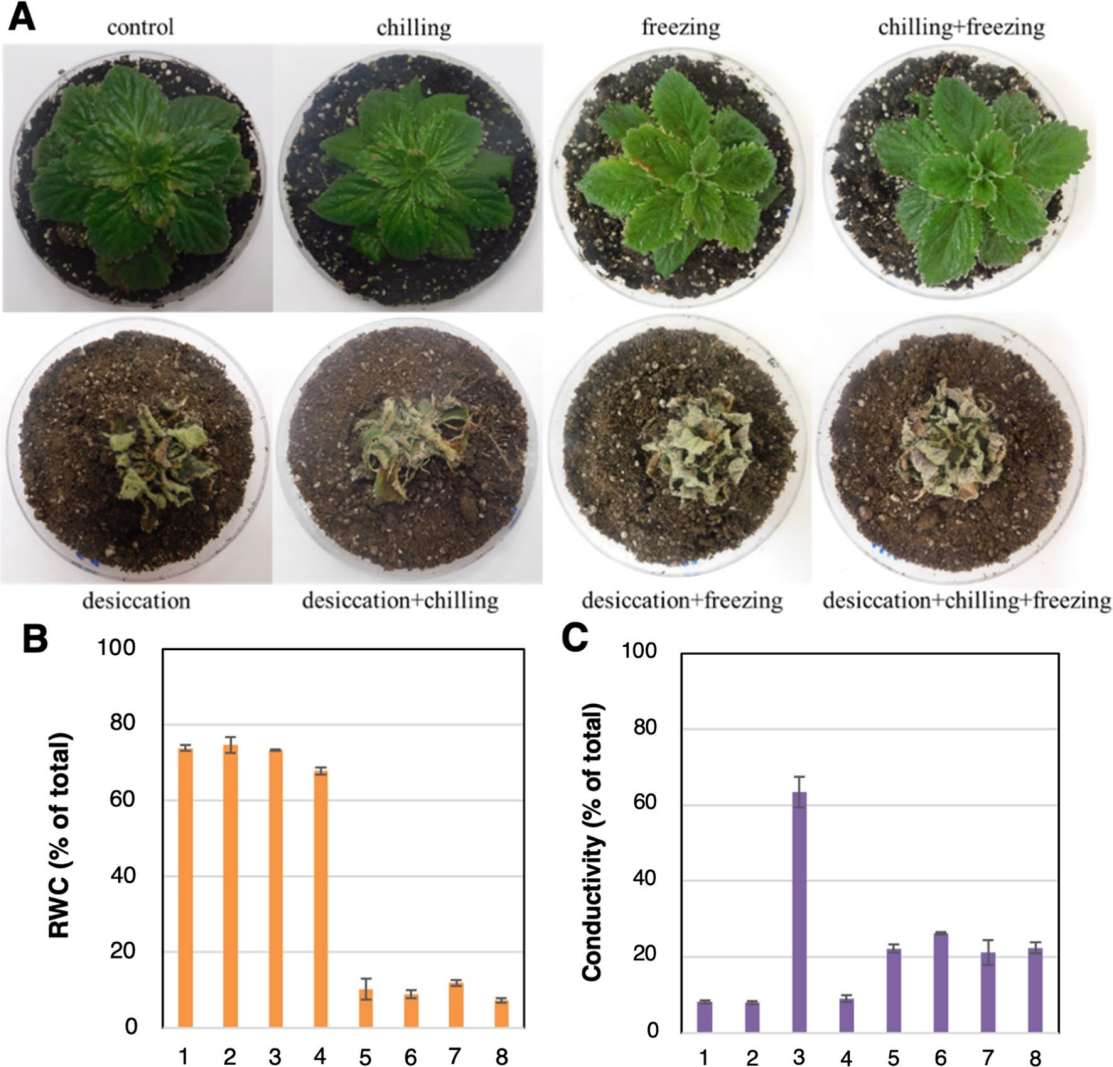
*H. rhodopensis* can tolerate low temperatures and desiccation. **A** Plants subjected to cold, freezing, desiccation, and their combination, as discussed in Materials and methods, at the end of the respective treatment and prior to sampling; **B** Relative water content; and **C** Electrolyte leakage measured as the conductivity of the respective plants. Data are means ± SD of three biological replicates. 1 – control; 2 – chilling; 3 – freezing; 4 – chilling and freezing; 5 – desiccation; 6 – desiccation and chilling; 7 – desiccation and freezing; 8 – desiccation, chilling and freezing

Next, we subjected *H. rhodopensis* plants to chilling (4 °C), freezing (− 4 °C), desiccation, and a combination of the stresses to inspect changes in their transcriptomes (see Materials and Methods for details). In well-watered and desiccation treated plants, chilling or freezing and their combination did not result in a noticeable change in phenotype and RWC (Fig. 4A–B). Interestingly, plants subjected to chilling did not show any cell damage (based on electrolyte leakage), whereas freezing induced significant cell damage (Fig. 4C). Notably, prior incubation at 4 °C for a day (chilling + freezing) completely rescued the observed rise of conductivity, indicating that this serves as an acclimation cue for the plants. A smaller but still significant elevation of electrolyte leakage, that was not further increased during freezing, was also detected in all desiccated plants. Thus, it appears that dried Haberlea plants exhibit enhanced tolerance to other abiotic stressors.

### Comparative transcriptome analysis of *H. rhodopensis* during desiccation, darkness, and their combination

**Fig. 5:**
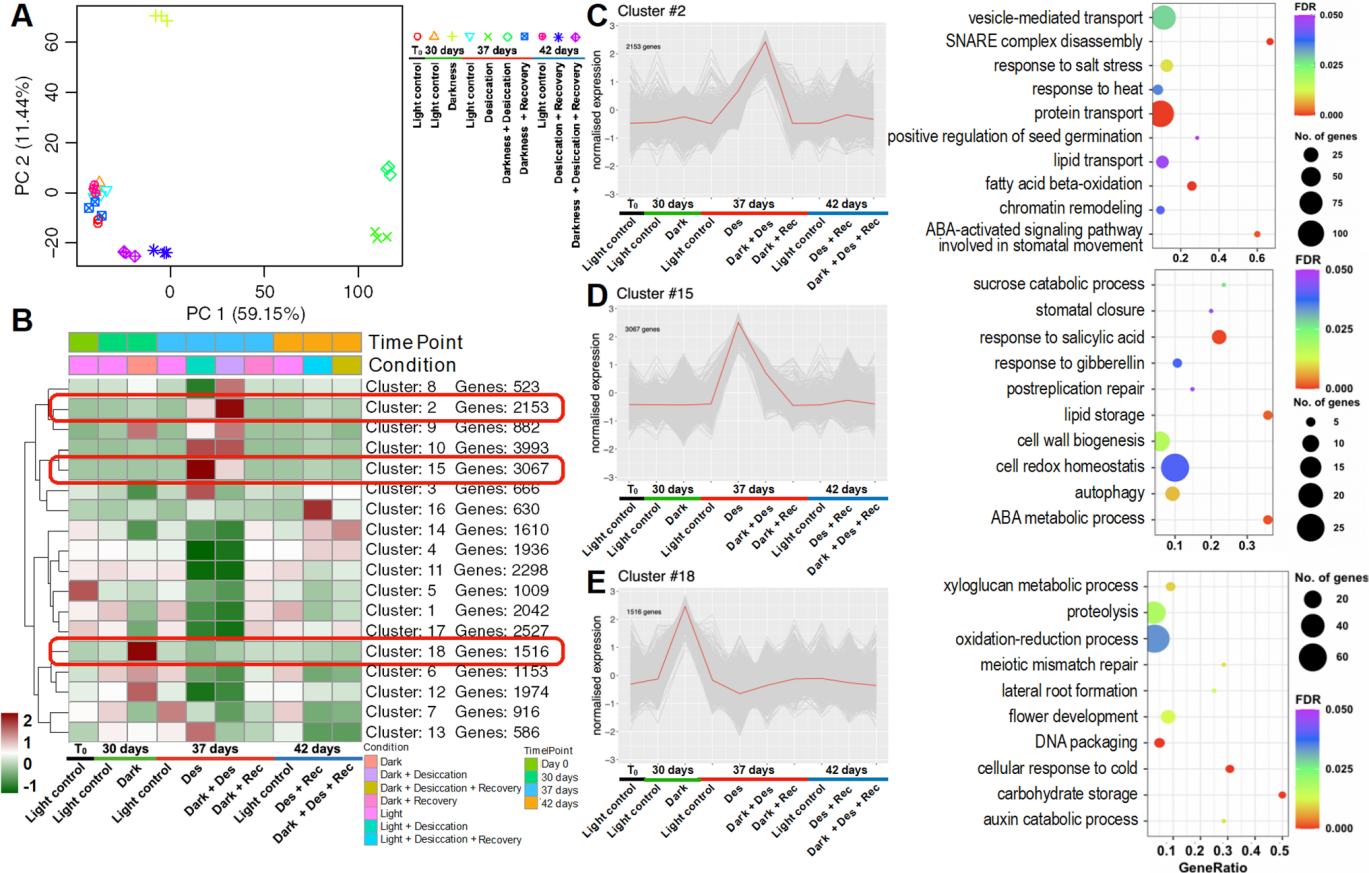
Cluster analysis depicting stress-specific gene expression across different time points for the darkness experiment. **A** Principal component analysis of the RNA-seq samples from the darkness experiment. Each symbol represents an individual sample. The three biological replicates are plotted using the same symbol. **B** K-means clustering of all differentially expressed genes. The number of clusters was determined using XMeans algorithm (RWeka package; [41]). The number of genes in each cluster is mentioned next to the cluster number. **C**, **D** and **E** correspond to clusters showing induced expression in desiccation in darkness (Cluster 2), desiccation (Cluster 15), and darkness (Cluster 18), respectively, along with a scatter plot of the selected significantly enriched GO terms (FDR < 0.05) of the genes present in respective clusters. Dot size corresponds to the differentially expressed genes (DEGs) with the respective GO term. GeneRatio represents the ratio of the number of DEGs annotated and the total number of genes annotated with the respective GO term. The color scale in (**B**) and the Y-axes in cluster plots in (**C**–**E**) represent the mean centered log2 normalized trimmed mean of *M* values (TMM) averaged across three biological replicates. The color scale in the scatter plot represents the FDR values. Abbreviations: Des, Desiccation; Rec, Recovery from respective stress. The complete lists of significantly enriched GO terms are provided in Data S2. The cluster numbers along with the list of DEGs are provided in Data S3

The total RNAs of leaves collected from plants subjected to darkness, desiccation, and the combination of the two stresses, and plants that have recovered from the three stress conditions, were sequenced in three biological replicates and compared to plants grown under control conditions (Fig. 3A). Principal component analysis (PCA) revealed different transcriptome profiles in plants from the stress conditions compared to control and recovered plants (Fig. 5A). Interestingly, desiccated samples and samples from the combined stresses closely grouped along PC1 (59.15%) and were different from the darkness samples, indicating that desiccation was a major contributor to the transcriptional changes compared to darkness (Fig. S4). All recovery samples exhibited transcriptome profiles similar to those of control samples, in concordance with the observed growth phenotypes.

We identified a total of 29,481 differentially expressed genes (DEGs) across all pairwise comparisons of conditions (Fig. S4; Data S3). The maximum DEGs were observed in desiccated samples and samples under combined stresses. Next, we clustered all DEGs using *k*-means clustering (*k* = 18) followed by GO and MapMan bin enrichment to identify affected biological processes (Data S2). The DEGs were grouped into 18 clusters based on their expression profiles (Fig. 5B). The clusters 2, 15, and 18 contained genes predominantly induced under desiccation in darkness, desiccation, and darkness, respectively (Fig. 5C–E). Cluster 2 mainly included genes related to ‘fatty acid beta-oxidation’, ‘chromatin remodeling’, ‘protein transport’, ‘ABA-activated signaling pathway involved in stomatal movement’, and ‘positive regulation of seed germination’. Genes predominantly induced during desiccation (cluster 15) were involved in ‘autophagy’, ‘stomatal closure’, ‘ABA metabolic process’, ‘postreplication repair’ and ‘lipid storage’. Dark stress resulted in up-regulation of genes (cluster 18) related to ‘carbohydrate storage’, ‘proteolysis’, ‘auxin catabolic process’, ‘lateral root formation’ and ‘regulation of flower development’.

Desiccation stress alone or in combination with darkness resulted in massive induction of genes encoding for ELIPs, heat shock proteins, such as HSP17.8, HSP17.9, HSP23.6, and HSP70, and enzymes related to sugar metabolism, such as STACHYOSE SYNTHASE and AMYLASES (ALPHA and BETA) (Data S3). Interestingly, 18KDa seed maturation protein was significantly induced (8.5-log_2_ fold change) under both desiccation and desiccation in darkness. Two more genes related to sugar transport, encoding the sucrose transport proteins SUC3 and SUC4, were induced exclusively by desiccation and desiccation in darkness. Late embryogenesis abundant (LEA) proteins, known to respond to dehydration, were substantially induced by desiccation regardless of the light regime, and a few *LEA* genes were also induced in darkness. Significant induction by desiccation, irrespective of the light regime, was also observed for several genes encoding signaling proteins, such as CBL-INTERACTING PROTEIN KINASE 2 and PROTEIN PHOSPHATASE 2C (PP2C).

On the other hand, genes encoding PHYTOCHROME INTERACTING FACTOR 1 (PIF1) and GROWTH RESPONSE FACTOR 4 (GRF4) were induced only in darkness and were unaltered by desiccation and desiccation in darkness (Data S3). Moreover, *GRF3* showed a rather contrasting expression pattern: it was significantly induced (3.6-log_2_ fold change) by darkness alone but repressed by desiccation or by desiccation in darkness. Similar expression behavior was observed for some phytochrome genes: *PHYTOCHROME A1* and *PHYTOCHROME B* were induced by darkness alone but repressed by desiccation and by desiccation in darkness. Contrasting expression patterns were also observed for several chlorophyll catabolism genes. One *PHEOPHYTINASE* gene was induced by desiccation and by desiccation in darkness but repressed by darkness. *STAY GREEN ONE* (*SGR1*)*, PHEOPHORBIDE A OXYGENASE* (*PAO*), and *RED CHLOROPHYLL CATABOLITE REDUCTASE* (*RCCR*) were induced by desiccation alone and by desiccation in darkness, but remained unaltered in the darkness alone.

The genes related to photosynthesis, including genes encoding CHLOROPHYLL *a*/*b* BINDING PROTEINS, LIGHT HARVESTING COMPLEX PROTEINS, and PROTOCHLOROPHYLLIDE REDUCTASE, and various other components of the photosynthetic machinery, were repressed by all stress conditions. The repression was the strongest by the combination of the two stress factors (desiccation in darkness), likely resulting in a complete photosynthesis shut-off.

**Fig. 6:**
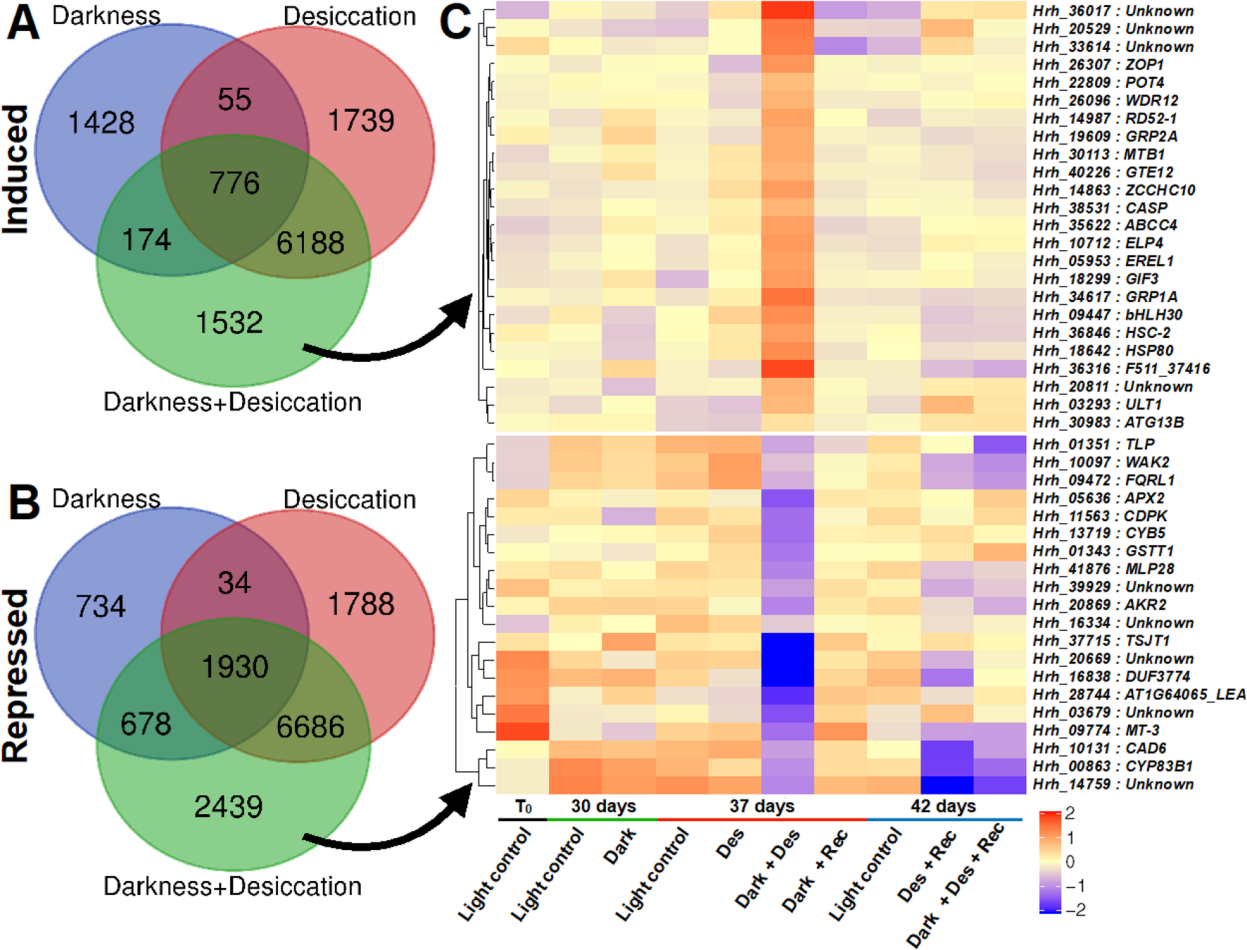
Overlap of genes induced and repressed at different stress conditions for the darkness experiment. **A**, **B** Venn diagram representing the number of specific and common differentially expressed genes (DEGs) induced (**A**) and repressed (**B**) across different stress conditions. The pairwise comparison of the stress condition with the respective developmental light control is used. **C** Expression profiles of selected genes specifically induced or repressed at desiccation in darkness. The color scales represent the mean centered log_2_ normalized trimmed mean of *M*-values (TMM) averaged across three biological replicates. The complete list of DEGs is provided in Data S3. Abbreviations: Des, Desiccation; Rec, Recovery from respective stress

Next, we investigated the DEGs affected by desiccation in darkness. We found 1532 genes specifically induced upon desiccation in darkness (Fig. 6A). Among these were genes encoding for transcription factors (bHLH30 and GTE12), glycine-rich RNA binding proteins (GRP1A and GRP2A), and autophagy-related protein 13b (ATG13B). Furthermore, 2439 genes including *ASCORBATE PEROXIDASE 2*, *GLUTATHIONE S-TRANSFERASE T1* (*GSTT1*), *CYTOCHROME P450 83B1* (*CYP83B1*), and a *LEA* gene were repressed upon desiccation in darkness only (Fig. 6B–C).

### Comparative transcriptome analysis of *H. rhodopensis* during chilling, freezing, desiccation, and combinations of these stress factors

**Fig. 7:**
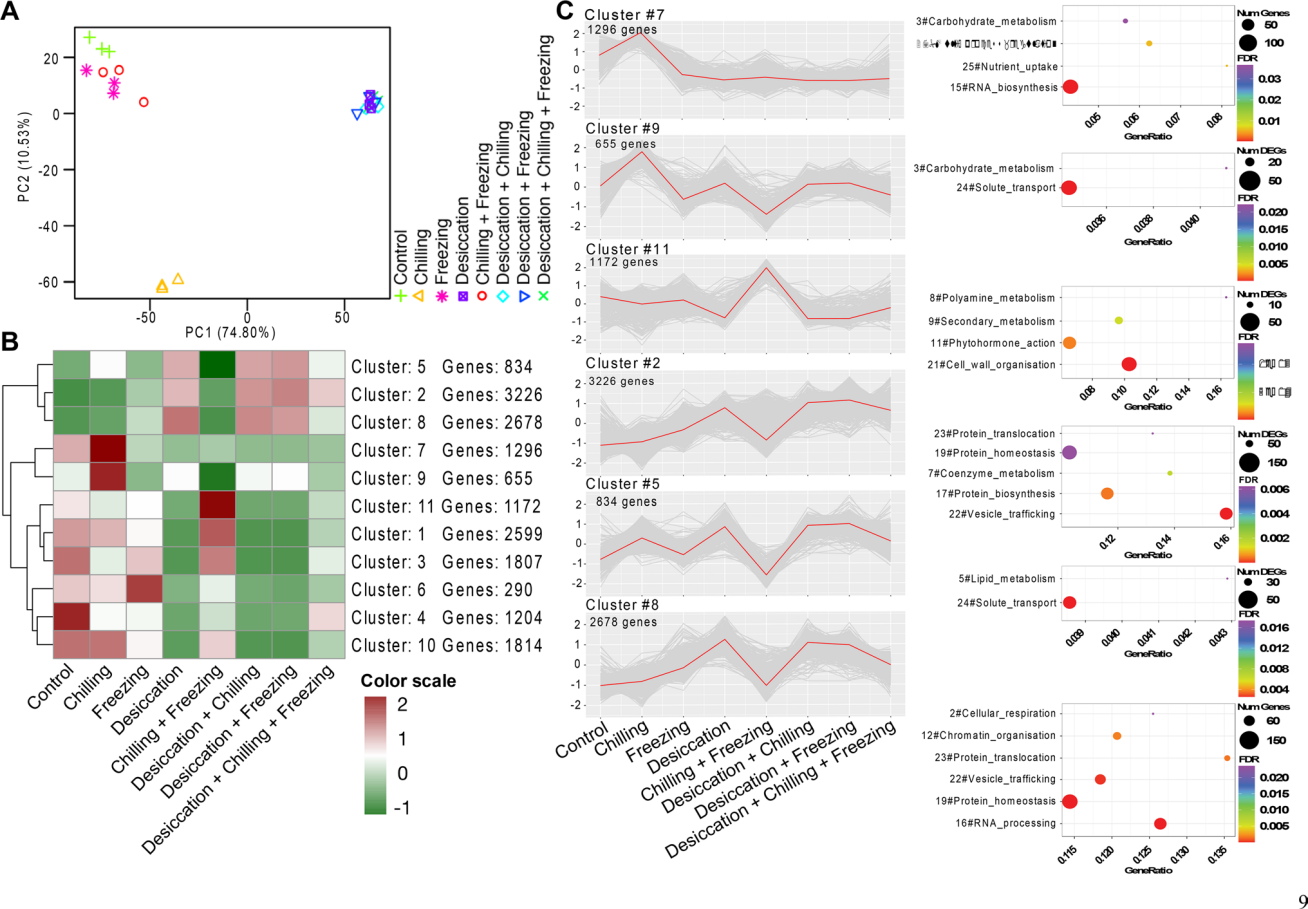
Cluster analysis depicting stress-specific gene expression across different time points for the cold experiment. **A** Principal component analysis of the RNA-seq samples. Each symbol represents an individual sample. The three biological replicates are plotted using the same symbol. **B** *K*-means clustering of all differentially expressed genes. The number of clusters was determined using XMeans algorithm (RWeka package). The number of genes in each cluster is mentioned next to the cluster number. **C** The line plots correspond to clusters showing induced expression upon chilling (Clusters 7 and 9), chilling + freezing (Cluster 11), and desiccation alone or in combination with other stresses (Clusters 2, 5 and 8). The scatter plots next to each line plot depict the enriched MapMan level 1 terms for the respective cluster. Dot size corresponds to the differentially expressed genes (DEGs) with the respective MapMan level 1 term. GeneRatio represents the ratio of the number of DEGs and the total number of genes annotated with the respective MapMan level 1. The color scale in (**B**) and *Y*-axis in cluster plots in (**C**) represent the mean centered log2 normalized trimmed mean of *M*-values (TMM) averaged across three biological replicates. The cluster numbers along with the list of DEGs are provided in Data S4. The complete lists of significantly enriched GO and MapMan terms are provided in Data S5

To study the transcriptional changes of *H. rhodopensis* plants subjected to low temperatures, the total RNAs from plants from the low-temperature experiment were sequenced (Fig. 7). In principle, the most dramatic reprogramming was induced by each of the four desiccation-related treatments, where ~ 6000 genes were upregulated, and ~ 8000 were downregulated (Figs. 7A and S6A–B; Data S4). Intriguingly, these sets of modulated genes overlapped to a very large extent, leaving relatively few condition-specific genes. This suggests that desiccation is the “master” stress factor and low temperatures cannot override it. Plants treated with freezing alone (followed by the combination of chilling and freezing) showed the lowest number of DEGs, whereas chilling alone resulted in much higher number of DEGs. Chilling, which in natural conditions is the first stressor that plants will experience in winter, may serve as a priming cue triggering a longer-lasting transcriptional reprogramming in order to prepare the plants for the subsequent sub-zero temperatures. Chilling and freezing do not share a large set of DEGs, pointing to distinct responses at the two temperatures.

In accordance with the small number of DEGs, freezing and chilling as well as freezing cluster relatively close with the controls (Fig. 7A). In turn, all desiccation-related samples form one supercluster, with a very well pronounced overlap, corroborating the observation above that low temperatures have negligible impact on dried Haberlea plants. The third group on the PCA, which is very well separated from the others, corresponds to the chilling stress and shows the unique characteristics of the Haberlea transcriptome in this condition, despite the lack of an observable growth phenotype.

Next, *k*-means clustering (*k* = 11) of the DEGs identified two clusters (clusters 7 and 9) consisting of genes specifically induced upon chilling (Data S4; Fig. 7B–C). Enrichment analysis suggested that these genes are involved in RNA biosynthesis, carbohydrate metabolism, nutrient uptake and multi-process regulation (Data S5). Cluster 11 was enriched for genes related to cell wall organization and phytohormone action; they were specifically induced by chilling and freezing (Fig. 7C). Clusters 2, 5, and 8 included genes encoding for signaling components that presumably act at the beginning of the signaling cascades and they were highly upregulated by desiccation, chilling, freezing, and the combinations of these stress factors (Fig. 7C). The genes included two PP2C protein phosphatases, whose homologs in *Arabidopsis* are known to be involved in ABA signaling, and a SRC2 homolog, which may act as an activator of the calcium-dependent activation of the NADPH oxidase RbohF that mediates reactive oxygen species (ROS) production (Data S5).

Several genes involved in selective protein degradation through the proteasome pathway, including the F-box stress-induced protein FBS1 and the E3 ubiquitin-protein ligase PUB18 that regulate ABA-mediated stomatal movement, were highly induced by dehydration, low temperatures, and the combination of these stresses (Data S4). At the same time, these stresses repressed the ubiquitin-protein ligase PUB23, known to negatively regulate water stress responses. The role of ABA was further substantiated by the induction of *LTI65* and *RCI2A* (Rare Cold-Inducible 2A) genes, known to be induced not only by ABA but also by low temperatures, dehydration, and salt stress [59–61]. The link between ABA signaling and protein degradation was confirmed by the induction of *EDL3* (*EID1-LIKE 3*) which encodes an F-box protein involved in mediating the regulation of abscisic acid signaling. EDL3 is known to regulate anthocyanin accumulation under drought stress [62]. Collectively, the data indicate roles of ABA signaling and protein degradation in both dehydration and low-temperature stress.

Transcription factor genes acting downstream in the gene regulatory networks were also significantly upregulated by all of these stress factors. These included *AZF2*, *DREB2A*, and *ZAT6*, reportedly involved in mediating the tolerance to other abiotic and oxidative stresses as well. These TFs in turn induce the expression of downstream stress-related genes that can contribute to the observed multiple stress tolerance. At the same time, other TF encoding genes, such as *ATHB-52*, *OFP4*, and *WRKY46*, were repressed by all stress factors studied. These TFs are likely to govern the stress-associated transcriptional reprogramming and confer multiple stress tolerance.

Genes encoding LEA proteins and ELIPs, acting further downstream the stress network, were among the most responsive in this experiment, and they were upregulated in all cases. Both families are typically associated with desiccation [63, 64], but here it is shown that some representatives are induced by low temperatures as well. The transcripts of some of the ELIPs, as well as LEA D-29, accumulate in all stress conditions, while the LEAs D-34 and SLE2 remain unchanged only during freezing and chilling + freezing. Interestingly, one of the LEAs–LEA 47, seems to be specific for low temperatures only since its transcription is not influenced by desiccation.

Two crucial enzymes involved in the synthesis of raffinose and raffinose family oligosaccharides (GOLS2 and RFS2) are considerably induced in all tested conditions. Thus, accumulation of raffinose family oligosaccharides (RFOs), previously associated mainly with desiccation, might be a universal stress response in Haberlea.

At the same time, genes involved in growth and development were repressed. These included *SAUR76*, encoding an auxin-responsive protein that promotes cell expansion, cell elongation, and plant growth in Arabidopsis [65], *HSD1*, involved in regulating plant growth and development by promoting or mediating brassinosteroid effects, and *EXPA2*, encoding an expansin. Many photosynthesis-related genes were repressed as well, indicating that inhibition of photosynthesis is a common response to all stresses.

In general, desiccation and chilling appear to inhibit auxin signaling and responses to auxin, as in the case of SAUR76, mentioned above, but also homologues of SAUR32 and SAUR50, the auxin-induced protein 15A (AX15A) and BIG-GRAIN 1-like B (BG3), involved in auxin transport. The situation with ABA is more complex, because while some aspects of the ABA pathways are induced, as EDL3 described above, others appear to be turned down in these conditions. For example, the receptor PYL4, known to inhibit the activity of group-A protein phosphatases type 2C (PP2Cs), is considerably downregulated. This is coupled to the measured hyperinduction of PP2Cs. In turn, abscisic acid 8'-hydroxylase 2, involved in the oxidative degradation of ABA, accumulated in all desiccated samples.

Somewhat unexpectedly, typical ROS scavengers are not among the most modulated in this experiment, which suggests that their expression is relatively stable, responsible for a constitutively active and highly effective antioxidant system. There are even some significantly downregulated ones during stresses: for example, the glutaredoxin GRXC13 and a peroxidase (PER42). Previously, it was suggested that *H. rhodopensis* might be preliminarily primed for drought/desiccation events [64], but this feature may be true for other abiotic stresses as well.

Among the most modulated genes, especially during desiccation and chilling, there is a large portion of cell wall-related ones. In most cases, they are downregulated—for example, expansins involved in cell expansion. The exception is a xyloglucan endotransglucosylase, which is induced by low temperatures and is normally associated with cell wall loosening by modification of the hemicellulose component.

### Comparative transcriptome analysis of *H. rhodopensis* subjected to different stresses

Next, we compared the DEGs from the darkness and low-temperature experiments to identify key genes commonly regulated by different stresses. For this, the DEGs were obtained using the pairwise comparisons of the stress time points against the respective controls (Fig. S7). We observed a significant overlap of the DEGs between the two experiments. The maximum overlap between the DEGs from the two experiments was observed for all time points involving desiccation stress, further suggesting that desiccation acts as a “master” stress factor. Furthermore, a total of 58 genes were either induced (51) or repressed (7) in all stresses, indicating these as common stress-responsive genes in Haberlea. Some of the commonly down-regulated DEGs included those encoding for expansins (*EXPA2* and *EXPA8*) and NAC TFs. Among the commonly up-regulated DEGs, eight encoded for TFs including members of NAC, MYB, C3H and C2H2 families.

### Transcription factors and resistance gene analogs

A total of 2507 transcription factors (TFs) belonging to 55 different families and representing 5.6% of the predicted protein-coding genes were identified in the *H. rhodopensis* genome (Table S7). TFs of the bHLH (218), MYB (195), ERF (174), and WRKY (151) families were the most abundant (Fig. S8A). The distribution of TF families is similar to those of other eukaryotes [37]. A significant number of the TFs (83.13%, 2084) were differentially expressed during different stages (Fig. S8B; Data S3 and S5) and 37 of these were specific to Haberlea.

**Fig. 8:**
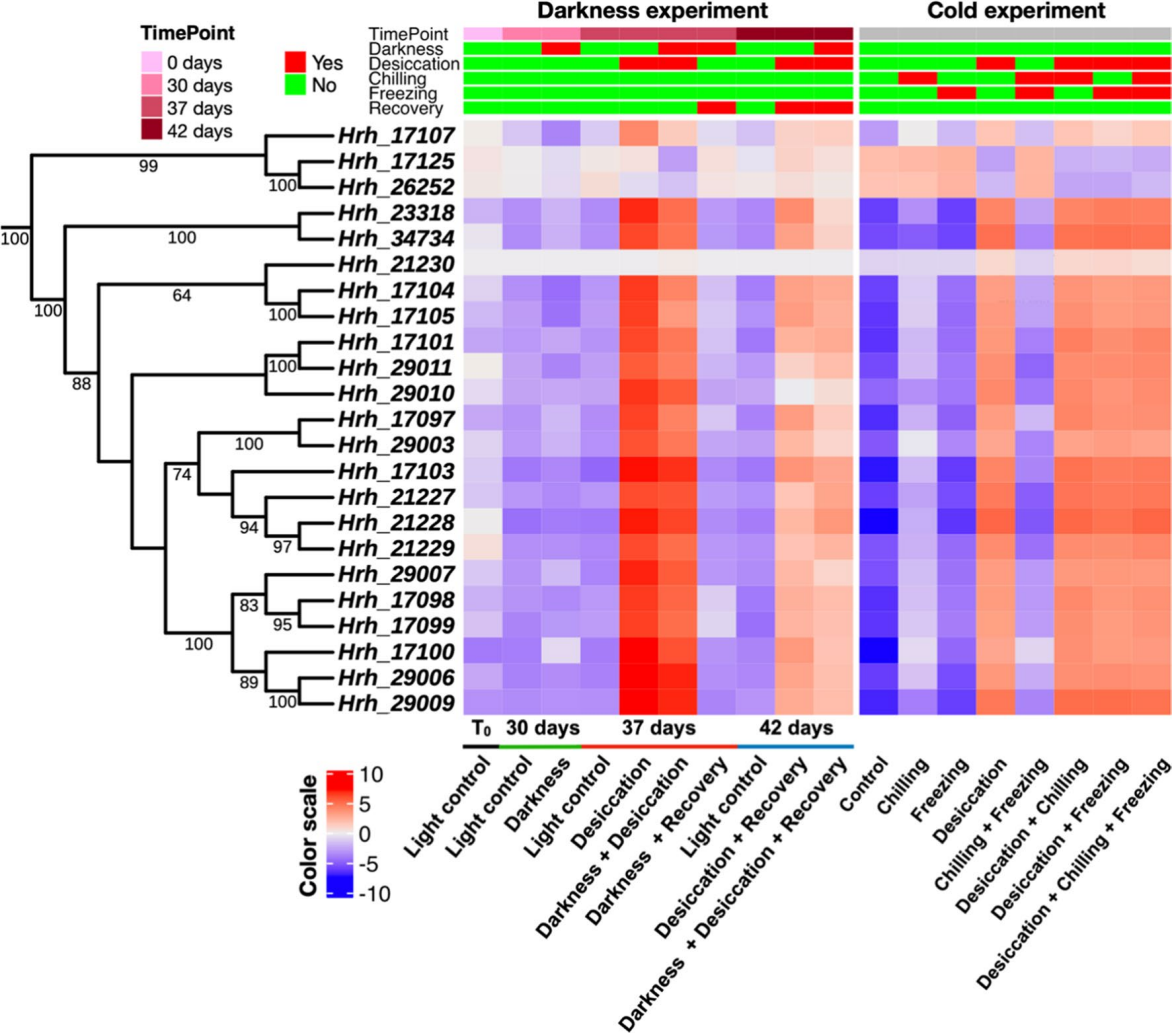
Expression profiles of *ELIPs* found in *H. rhodopensis*. The phylogenetic tree depicts the clustering of protein sequences of all *ELIP* genes found in the *H. rhodopensis* genome. The expression of the *ELIP* genes in all samples is plotted as a heatmap. The color scale represents the mean centered log_2_ normalized trimmed mean of *M* values (TMM) averaged across three biological replicates

Resistance gene analogs (RGAs) are activated by various biotic and abiotic stresses [66]. We predicted a total of 873 RGAs in the genome of *H. rhodopensis*. These RGAs included 102 NBS-encoding proteins, 51 receptor-like proteins (RLPs), 575 receptor-like kinases (RLKs), and 145 transmembrane coiled-coil (TM-CC) proteins (Table S8). Out of the 102 NBS-encoding genes, 77 (75.49%) were differentially expressed in one of the pairwise comparisons (Fig. S9) and two of these were Haberlea-specific. Considering the importance of ELIPs in desiccation tolerance, we investigated these genes in the *H. rhodopensis* genome. Using similarity searches with other plant species, we identified a total of 23 ELIPs, arranged in tandem arrays across the genome (Fig. 8). All *ELIPs* were differentially expressed in at least one pairwise comparison with 20 *ELIPs* being significantly induced by desiccation alone or in combination with other stresses.

## Discussion

### Haberlea can withstand a combination of abiotic stresses

Here we showed that the desiccation tolerance in *H. rhodopensis* is fully preserved in darkness, as well as in chilling/freezing temperatures. Prolonged darkness combined with low temperatures occurs, for example, around and beyond the polar circles. Air-dried plants grown under photoperiod or in complete darkness withstand desiccation to just 5% RWC, which would otherwise kill non-resurrection plants. Measurements of the electrolyte leakage confirmed that neither the two stresses alone nor their combination inflicted serious damage on the plants. The full recovery of *Haberlea rhodopensis* from full and fast air-drying seems a special feature for this species, as the recovery from dehydration of other resurrection species, such as *Boea hygrometrica* and *Craterostigma plantagineum*, is more problematic after fast air-drying. Additionally, we demonstrated that *H. rhodopensis* is tolerant to chilling and once in a dehydrated state, it can also tolerate freezing (sub-zero) temperatures.

### Haberlea-specific genes and expansion of gene families involved in stress tolerance

Here, we report a high-quality sequencing and assembly of the *H. rhodopensis* genome, whose size (~ 1.27 Gb) is similar to the genome of its close relative *B. hygrometrica* [46]. We identified 10,435 genes specific for Haberlea (not sharing sequence homology with other species), constituting a very high percentage of the predicted genes (23.55%). Genes specific to only one or a few closely related species are known as taxonomically restricted genes (TRGs) [67]. TRGs are implicated in the adaptation to unfavorable environments [68]. It is likely that some of the TRGs of *H. rhodopensis* are important for the tolerance to extreme abiotic stress factors, such as desiccation, darkness, and chilling/freezing. Many genes specific to *H. rhodopensis* encode proteins with unknown functions. Future functional studies may reveal new and important players in the adaptation to extreme environments.

Expansion of particular gene families is related with their increased importance for adaptation to new or/and extreme environments. The large number of gene families expanded in *H. rhodopensis* (89 in total) supports this notion. Members of some of these gene families are known to be involved in responses to abiotic factors, such as drought/desiccation and photoperiod (light/darkness). For example, a *SERINE/THREONINE-PROTEIN PHOSPHATASE*, *DISEASE RESISTANCE PROTEINs*, and two *ELIP* genes were the highest induced genes in Haberlea exposed to desiccation [1]. The *ELIP* family was shown to have expanded in all resurrection species [56]. Protein phosphatase 7 (PP7) proteins are specifically present in the plant kingdom and are localized mainly in the nucleus [69]. In *Arabidopsis*, AtPP7L is involved in chloroplast development, and its overexpression confers resistance to highlight stress [70]. In *H. rhodopensis*, we found a significant expansion of the *SERINE/THREONINE-PROTEIN PHOSPHATASE 7 LONG FORM* family. The gene families expanded in *H. rhodopensis* are also involved in pathways such as the SnRK1 kinase regulatory system, known for its role in adjusting cellular metabolism during starvation and stress conditions [71]. Furthermore, in the recently sequenced cold-tolerant extremophile *Eutrema salsugineum* [72], the expanded gene families were enriched for genes related to cold response and hormone signaling which was also the case in *H. rhodopensis*, suggesting similarities in adaptation to cold.

### Transcriptional rewiring during desiccation in darkness

**Fig. 9:**
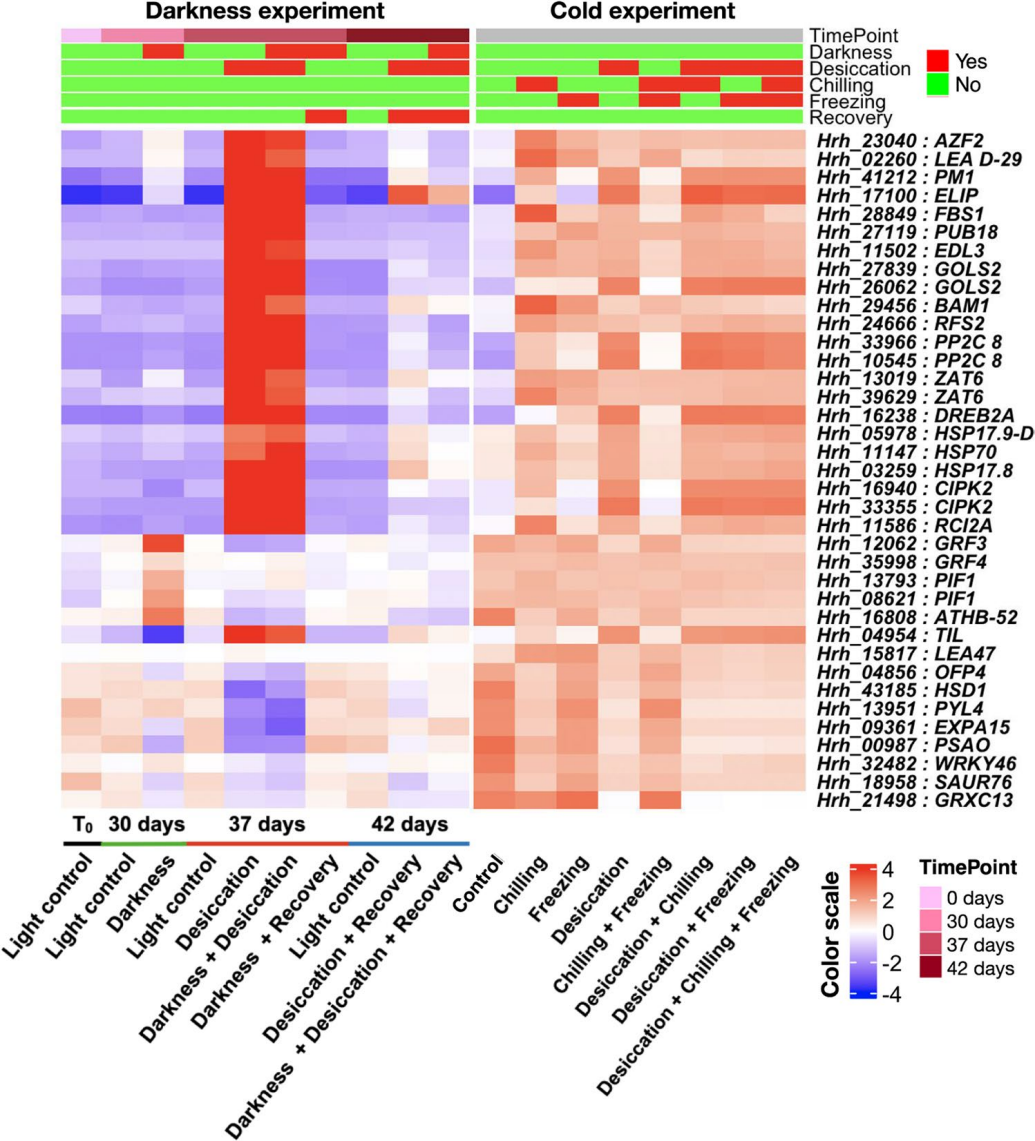
Expression profiles of key *H. rhodopensis* genes of interest, selected among the most modulated in response to one or more of the studied stresses. The color scales represent the mean centered log2 normalized trimmed mean of *M*-values (TMM) averaged across three biological replicates. The complete list of DEGs is provided in Data S3 and S4

Very low light and even darkness can occur in different habitats, for example in the undergrowth under dense forests or around the polar circles during the long winters. *H. rhodopensis* is the only species with a sequenced genome that can withstand extended darkness. Furthermore, it is one of the very few species in which comprehensive molecular studies on adaptation to darkness have been performed. Overall, transcriptional regulation during desiccation was more prominent than during darkness. Some of the key genes are highlighted in Fig. 9. The massive induction of *ELIP*, *LEA*, and *HSP* genes in the desiccated Haberlea plants confirmed earlier studies showing that these genes are induced by dehydration in vegetative tissues of both model and resurrection species, including *H. rhodopensis* [1, 73]. The *ELIP* gene family, as noted above, expanded in all resurrection plants, including Haberlea, and the *ELIP* genes are switched on during desiccation and light stress to protect against these abiotic stresses [55, 56]. However, as seen in Fig. 8, our results demonstrate that the expression of the majority of *ELIP* genes is significantly upregulated also during desiccation in the dark. This suggests that ELIPs are an indispensable component of the desiccation response and that their role is not limited to the presence of light. Moreover, it appears that there is a bypassing mechanism, which, upon desiccation, activates the expression of *ELIPs* and other light-related genes independently of light, as their usual stimulus. LEAs are known to respond to dehydration in drying seeds and desiccated vegetative tissues. However, some *LEA* genes in Haberlea (*LEA 2* and *LEA D-29*) are highly induced by darkness as well suggesting that they are highly important for plant survival in darkness [3]. Interestingly, one of the *LEA* genes (*AT1G64065_LEA*, Fig. 8) was specifically repressed upon desiccation in darkness.

Sugars are implicated in the defense against dehydration in many ways: accumulation of sucrose as water replacement is a universal response to desiccation of many resurrection species, and raffinose family oligosaccharides (RFOs), such as raffinose, stachyose, and verbascose, can protect against drought-induced oxidative stress [1, 74–76]. In line with these previous studies, it was not surprising to see that many genes related to sugar metabolism and transport respond to desiccation in Haberlea. The induction of stachyose synthase may contribute to the accumulation of RFOs, whereas the induction of the sucrose transport protein genes *SUC3* and *SUC4* may contribute to the required increase in sucrose mobility during the stress response [1, 77, 78].

Several genes encoding for signaling proteins seem to be specific for drought/desiccation. These include a *CBL-INTERACTING PROTEIN KINASE* 2, a *PROTEIN PHOSPHATASE 1 REGULATORY SUBUNIT*, and a *PP2C* gene. The *PP2C* gene is also the highest induced gene at both the earliest and the latest time points during dehydration [1]. This, together with its presumed function as a component situated at the beginning of the signaling cascade, makes it an ideal candidate for further functional studies.

Moreover, in *H. rhodopensis*, we found upregulation of several bHLH encoding genes during desiccation and desiccation in darkness. On the other hand, *GRF3*, *GRF4*, and *PIF1* are specific for darkness. In *Arabidopsis*, GRF3 is implicated in integrating environmental stimuli into developmental programs: genes downstream of GRF3 are related to plant growth, development, phytohormone biosynthesis and signaling, and the cell cycle [79]. In rice, GRF4 promotes and integrates nitrogen assimilation, carbon fixation, and growth [80]. Regulation of growth is certainly critical in Haberlea exposed to long-term darkness. Darkness and desiccation activate the autophagy pathway, as several genes specific for autophagy were upregulated in the absence of light and/or in dehydrated samples. This seems to reflect the starvation specifically caused by darkness or/and desiccation, as the low temperatures alone did not upregulate these autophagy-related genes.

### Transcriptional reprogramming at low temperatures

The RNA seq analysis of the samples from the low-temperature experiment indicated that chilling and freezing induce a very dissimilar transcriptomic response in *H. rhodopensis*. Many of the genes typically associated with desiccation tolerance, such as *ELIPs* and *LEAs*, and representatives of signal transduction pathways, such as F-box proteins and *EARLY RESPONSIVE TO DEHYDRATION 7* (*ERD7*), were also induced by low temperatures, especially chilling. Conversely, other genes mostly associated with cold responses were upregulated by desiccation as well. This includes *RARE COLD-INDUCIBLE 2A* (*RCI2A*), *COLD REGULATED GENE 27* (*COR27*), a temperature-induced lipocalin (*TIL*), and a low temperature-induced 65 kDa protein (*LTI65*). Additionally, several TFs known to regulate cold tolerance including INDUCER OF CBF EXPRESSION 1 (*ICE1*), and *DREB3* were induced in low-temperature conditions. Interestingly, a DREB3 homolog and a bHLH-domain containing protein were induced only during all low-temperature time points. The bHLH and DREB TFs are also shown to be induced in cold-tolerant halophyte (*E. salsugineum* [81]). Furthermore, in transgenic tomato *DREB3* overexpression improves tolerance to cold stress [82]. This shows that *H. rhodopensis* utilizes the same protective strategy elements for different purposes. In turn, genes related to multiple aspects of photosynthesis are always downregulated in response to these stressors, demonstrating that photosynthesis shutdown is a standard reaction against abiotic challenges.

Overall, by combining genome assembly and transcriptome analyses, the study presented here reveals some of the important players, such as *PP2C*, *ERD7*, and *TIL*, involved in the responses of *H. rhodopensis* to several different abiotic stresses. These are suitable candidates for future functional studies and are a valuable resource for the scientific community that enables a better understanding of mechanistic aspects of desiccation and the response to long-term darkness and low temperatures in plants. The information obtained by dissecting the molecular responses of different stresses in naturally tolerant plants, like key genes and/or genetic variants, as well as specific regulation mechanisms, can be translated into designing strategies for increasing stress resistance in other food crops. For example, factors simultaneously contributing to desiccation tolerance in vegetative tissues in resurrection species and in dehydrating seeds in crops are promising candidates for that purpose. Genes from desiccation-tolerant species have already been shown to function in model plants and crops and mitigate abiotic stresses, such as drought and salinity [83, 84]. Identifying novel Haberlea genes and pathways that potentially contribute to tolerance to extreme environments may also pave the way for engineering crops with multiple stress tolerance and higher productivity under unfavorable conditions.

### Supplementary Information

Below is the link to the electronic supplementary material.

Supplementary file1 (XLSX 127 KB)

Supplementary file2 (DOCX 12292 KB)

Supplementary file3 (XLSX 10 KB)

Supplementary file4 (XLSX 109 KB)

Supplementary file5 (XLSX 9735 KB)

Supplementary file6 (XLSX 6143 KB)

Supplementary file7 (XLSX 62 KB)

## Data Availability

Genome and transcriptome sequencing data, and genome assembly and annotation from this article can be found in the EBI ENA database under accession number PRJEB40105.
