# Supplementary material for: The genome of Haberlea rhodopensis provides insights into the mechanisms for tolerance to multiple extreme environments
Source: Cell Mol Life Sci. 2024 Mar 5;81(1):117. doi: 10.1007/s00018-024-05140-3 (PMC10914886; doi:10.1007/s00018-024-05140-3)
Supplement: Supplementary file 2 — Supplementary file2 (DOCX 12292 KB) [file 18_2024_5140_MOESM2_ESM.docx]

**Supplementary Figures S1-S9**

**
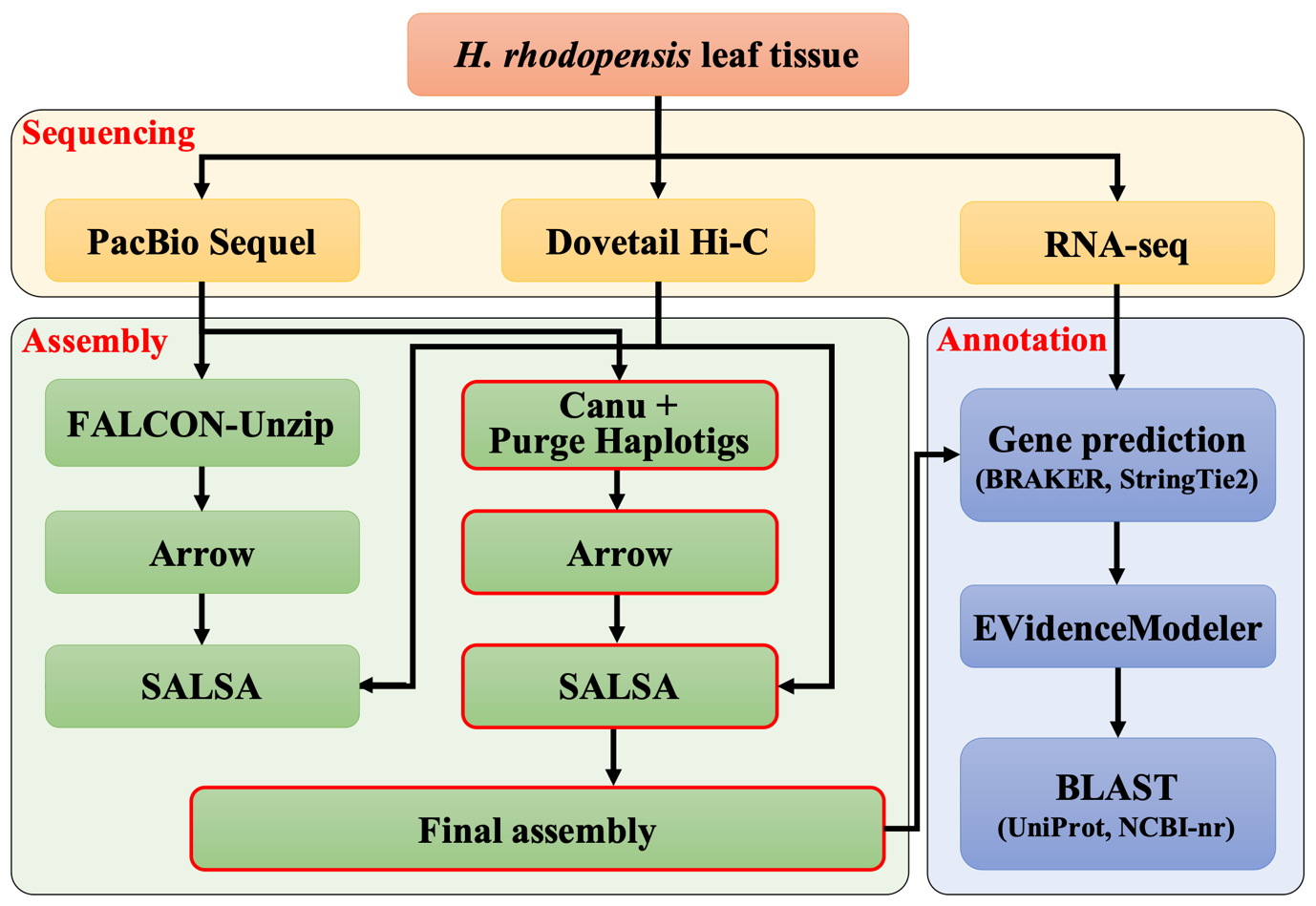
**

**Figure S1. Workflow for sequencing, assembly and annotation of the *H. rhodopensis* genome.** The boxes in red border indicate the steps leading to the final assembly (Canu and Purge Haplotigs, followed by Arrow and SALSA) used for downstream analysis.

**B**

**A**

**Figure S2. GC content and Ks distribution.** (A) The GC content was calculated for the genomes of *H. rhodopensis* (38.1%), *B. hygrometrica* (42.3%), *X. viscosa* (36.5%), *S. lycopersicum* (34.0%), *A. thaliana* (36.0%), *C. plantagineum* (40.2%), *L. brevidens* (39.3%), and *L. subracemosa* (38.7%) using 50-kb non-overlapping sliding windows. (B) Distribution of ks values of the paralogous pairs identified in *H. rhodopensis*, *A. thaliana*, and *S. lycopersicum*. The blue arrows indicate the duplication events in *H. rhodopensis.*


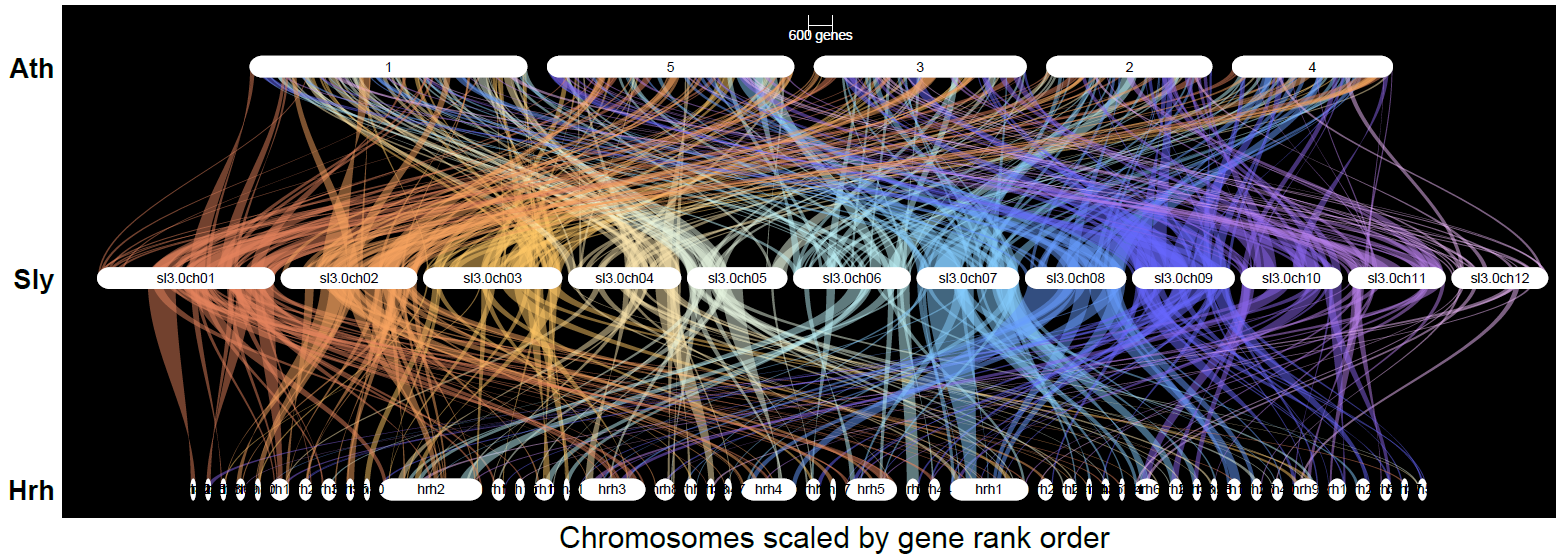


**Figure S3. Synteny analysis of *H. rhodopensis* with *A. thaliana* and *S. lycopersicum*.** The syntenic map (‘riparian plot’) of orthologous regions among *H. rhodopensis* (Hrh), *A. thaliana* (Ath) and *S. lycopersicum* (Sly). The ribbons are color coded by synteny to *S. lycopersicum* chromosomes. Scaffold segment sizes are scaled by the total number of genes in syntenic networks and positions of the braids are the gene order along the sequence.

**Figure S4. MapMan bin enrichment of gene families expanded in *H. rhodopensis*.** Scatter plot shows the significantly (FDR < 0.05) enriched MapMan4 level 2 bins. Dot size corresponds to the number of genes. GeneRatio represents the ratio of the number of genes in the MapMan bin and the total number of genes with annotation in the respective MapMan bin.

**Figure S5. Differential expression analysis for darkness experiment.** Number of differentially expressed genes (DEGs) for all pairwise combinations from the darkness experiment. The DEGs were determined using |log_2_ fold change| ≥ 1 and FDR < 0.05.

**B**

**A**

**Figure S6. Differential expression analysis for cold experiment.** (A) UpSet plot depicting overlap of up-regulated DEGs. (B) UpSet plot depicting overlap of down-regulated DEGs. The DEGs were determined using |log_2_ fold change| ≥ 1 and FDR < 0.05. Abbreviations: Des, desiccation; Chl, chilling; Fre, freezing.


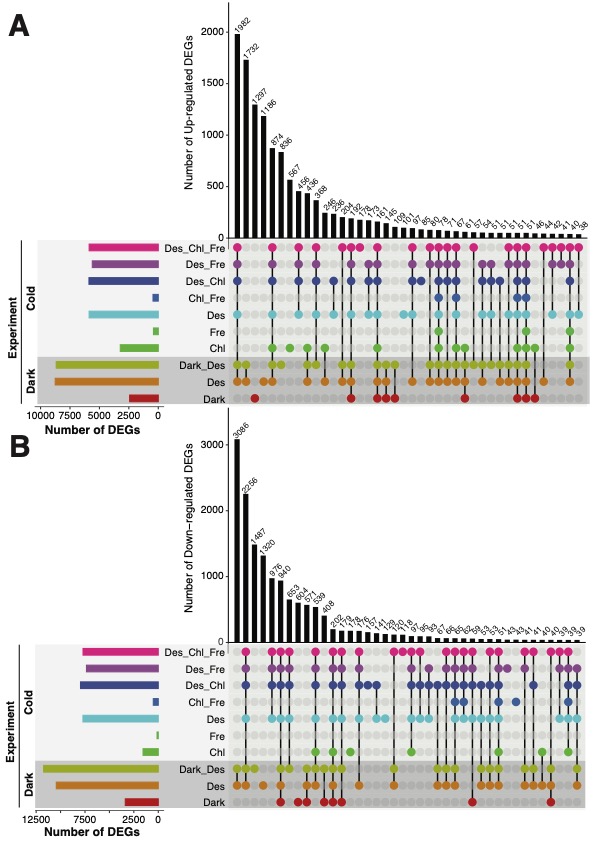


**B**

**A**

**Figure S7. Overlap of differentially expressed genes between cold and darkness experiments.** (A) UpSet plot depicting overlap of up-regulated DEGs. (B) UpSet plot depicting overlap of down-regulated DEGs. The pairwise comparisons of the stress time point with the respective control were used for identifying the overlapping DEGs. The horizontal bars (left) show the total number of DEGs for each pairwise comparison; vertical bars represent the frequency for each intersection (shared or unique DEGs), and colored circles highlight the pairwise comparison that are part of the intersection. Abbreviations: Des, desiccation; Chl, chilling; Fre, freezing.

**B**

**A**

**Figure S8. Distribution and expression of transcription factors in *H. rhodopensis*.** (A) The bar plot depicts the 15 most abundant transcription factor (TF) families. (B) The heatmap shows the expression profiles of differentially expressed TFs from 10 most abundant families. The color scale represents the mean centered log_2_ normalised trimmed mean of M-values (TMM) averaged across three biological replicates.

**Figure S9. Differentially expressed resistance gene analogs (RGAs).** The genes are grouped according to the different sub-classes of RGAs. The color scale represents the mean centered log_2_ normalised trimmed mean of M-values (TMM) averaged across three biological replicates. Abbreviations: NBS, nucleotide-binding site containing protein; RLK, receptor-like protein kinase; RLP, receptor-like protein; TM-CC, transmembrane coiled-coil protein.
